# Supplementary material for: Health aspects and lifestyle of licensed manual therapists during the COVID-19 pandemic in Sweden: The CAMP cohort study
Source: PLoS One. 2025 Oct 7;20(10):e0327600. doi: 10.1371/journal.pone.0327600 (PMC12503269; doi:10.1371/journal.pone.0327600)
Supplement: S1 File — (DOCX) [file pone.0327600.s001.docx]

| **Table S1.** What have you done to promote your own health during the COVID-19 pandemic? (Baseline) | | |
| --- | --- | --- |
| **n = 664^a^ Total number of counts = 1645^b^** | | |
| **Subcategory (number of counts)** | **Category** | **Theme** |
| Exercise (508) | Physical activity | Physical health |
| Healthy diet (163)  Reduced alcohol consumption (4)  supplements/micronutrients (92) | Diet |  |
| Sleep habits (101) | Sleep |  |
| Lifestyle habits (2)  Maintain healthy routines (20) | Routines |  |
| Cold-water immersion/sauna (9)  Fasting (4)  Immune boosting (8)  Chiropractic treatment (13) | Alternative methods |  |
|  |  |  |
| Enjoyable activities (15)  Watch movies/Read books (4)  Gotten a dog (1)  Spend time with family (46)  Keep social contacts (3) | Health promotion | Mental health |
| Outdoors/nature (186)  Bicycle commuting (22)  Change environment (1) | Physical environment |  |
| Recovery routines/Stress  management/relaxation/meditation (42)  Positive thinking (13)  Avoid stress (13)  Pacing/regular breaks/Less work/listen to the body (18)  Avoid news (3) | Stress management |  |
|  |  |  |
| Avoid crowded settings/unnecessary contacts (66)  Hygiene (86)  Avoid public transportation (25)  Follow recommendations (40)  Social distancing (46)  Stay at home with symptoms (5)  Staying home (2)  Reduced travel (2)  Avoiding handshakes or hugs (3)  Read information regarding the pandemic (2)  Socialize with colleagues (6)  Isolation (3)  Communicate/socialize in new ways (21)  Limited social life (47) | Recommendations | Follow Recommendations |
| ^a^ Number of participants answering the question.  ^b^ The total number of codes categorized into different subcategories. Each participant’s response could generate several codes and contribute to multiple subcategories forming each category. One category could therefore comprise a higher number of counts than the total number of participants answering the question. | | |

| **Table S2.** What have you done to promote your own health the past six months? (12-month follow-up) | | |
| --- | --- | --- |
| **n = 486^a^ Total number of counts = 1026^b^** | | |
| **Subcategory (number of counts)** | **Category** | **Theme** |
| Exercise (432) | Physical activity | Physical Health |
| Healthy diet (163)  Supplements/micronutrients (40)  Reduced alcohol consumption (2) | Diet |  |
| Sleep habits (90) | Sleep |  |
| Maintain healthy routines (20)  Manage asthma medications (1)  Work (2) | Routines |  |
| Cold-water immersion (1)  Fasting (2)  Chiropractic treatment (6) | Alternative methods |  |
|  |  |  |
| Enjoyable activities (14)  Watch movies/read books (3)  Gotten a dog (1)  Spend time with family (29)  Keep social contacts (32) | Health promotion | Mental health |
| Outdoors/nature (42)  Bicycle commuting (5)  Change environment (2)  Study/Sell clinic (2) | Physical environment |  |
| Recovery routines/stress management/relaxation/meditation (61)  Positive thinking (2)  Avoid stress (4)  Pacing/regular breaks/less work/listen to the body (5)  Avoid news and social media (3)  Sick leave (1) | Stress management |  |
|  |  |  |
| Avoid crowded settings/unnecessary contacts (8)  Hygiene (8)  Follow recommendations (3)  Social distancing (7)  Stay at home with symptoms (1)  Isolation (32)  COVID-19 vaccine (2) | Recommendations | Follow recommendations |
| ^a^ Number of participants answering the question.  ^b^ The total number of codes categorized into different subcategories. Each participant’s response could generate several codes and contribute to multiple subcategories forming each category. One category could therefore comprise a higher number of counts than the total number of participants answering the question. | | |

| **Table S3.** Comments regarding how the COVID-19 impacted your physical health (Baseline) | | |
| --- | --- | --- |
| **n = 202^a^ Total number of counts = 342^b^** | | |
| **Subcategory (number of counts)** | **Category** | **Theme** |
| Less exercise (62)  Avoid gym or other training facilities (34)  Reduced cardiorespiratory fitness (5)  More sedentary time (4)  Reduced strength (4)  Reduced flexibility (2) | Impaired physical fitness | Negative consequences of the pandemic |
| Worse sleep (5)  Increased alcohol consumption (1)  Weight gain (5)  Bad diet (3)  Less leisure time (1) | Impaired lifestyle |  |
| Fear/worry (14)  Boredom (2)  Stress (16)  Fatigue (10)  Shame (1)  Uncertainty (1)  Worse mental well-being (2)  Hypochondria (1)  Increased family burden (2)  Economic stress (4) | Mental health consequences |  |
| Infections (6)  COVID-19 (28)  Long COVID (13) | COVID and other infections |  |
| Consequences of disease (3)  Sick leave (4)  Musculoskeletal pain (8)  Headache (4)  Side effects of sanitation (1)  Weightloss (2) | Ill health |  |
|  |  |  |
| Fewer infections (19)  No patients with infections (4) | Fewer infections | Positive consequences of the pandemic |
| More exercise (25)  Increased strength (1)  More everyday physical activity (6) | More exercise |  |
| More leisure time (5)  More time in nature (1)  Better sleep (1)  Better diet (2)  Reduced stress (2)  More calm (1)  Supplements/micronutrients  (2)  Less musculoskeletal pain (1) | Improved lifestyle |  |
|  |  |  |
| Exercising alone (2)  Changed exercise time (2)  Outdoor training (2)  Less indoor training (1)  Changed exercise (2) | Change in exercise | Changes due to recommendations |
| Less social contacts (4)  Social distancing (3) | Social distancing |  |
| Less physical work (1)  Less work (3) | Less work |  |
| More physical work (1)  Hygiene (2) | More work |  |
| ^a^ Number of participants answering the question.  ^b^ The total number of codes categorized into different subcategories. Each participant’s response could generate several codes and contribute to multiple subcategories forming each category. One category could therefore comprise a higher number of counts than the total number of participants answering the question. | | |

| **Table S4.** Comments regarding how the COVID-19 impacted your physical health the last six months (12-month follow-up) | | |
| --- | --- | --- |
| **n = 99^a^**  **Total number of counts = 147^b^** | | |
| **Subcategory (number of counts)** | **Category** | **Theme** |
| Less exercise (25)  Avoid gym or other training facilities (5)  Reduced cardiorespiratory fitness (1)  Worse physical fitness (1)  Reduced everyday physical activity (1) | Impaired physical fitness | Negative consequences of the pandemic |
| Worse sleep (1)  Weight gain (7) | Impaired lifestyle |  |
| Fear/worry (2)  Stress (4)  Fatigue (6)  Economic stress (3) | Mental health consequences |  |
| Infections (4)  COVID-19 (14)  Long COVID (11)  Side effects of COVID-19 vaccine (5) | COVID and other infections |  |
| Musculoskeletal pain (7)  Weight loss (1)  Side effects of personal protective equipment (4) | Ill-health |  |
|  |  |  |
| Fewer infections (5) | Fewer infections | Positive consequences of the pandemic |
| More exercise (13)  Increased strength (1)  More everyday physical activity (1) | More exercise |  |
| Better sleep (2)  More calm (2)  Supplements/micronutrients  (1)  Less musculoskeletal pain (1) | Improved lifestyle |  |
|  |  |  |
| Outdoor exercise (2) | Changed training | Changes due to recommendations |
| Less social contacts (3)  Social distancing (1) | Social distancing |  |
| Less physical work (1)  Less work (2) | Less work |  |
| More physical work (1)  More job satisfaction (2) | More work |  |
| ^a^ Number of participants answering the question.  ^b^ The total number of codes categorized into different subcategories. Each participant’s response could generate several codes and contribute to multiple subcategories forming each category. One category could therefore comprise a higher number of counts than the total number of participants answering the question. | | |

| **Table S5.** Comments regarding how your impacted physical health due to the COVID-19 pandemic affected your work ability (Baseline) | | |
| --- | --- | --- |
| **n = 74^a^**  **Total number of counts = 99^b^** | | |
| **Subcategory (number of counts)** | **Category** | **Theme** |
| Less exercise (3)  Reduced strength (2)  Less energy (17) | Impaired physical ability | Changed physical ability |
| More energy/strength (9)  Happier (1)  Healthier (3) | Improved physical ability |  |
|  |  |  |
| More work (5)  More patients seen per workday (2)  Physically heavier work (1)  Stress (6) | Increased workload | Changed workload |
| Less physical work (7)  Less work (8)  Flexibility (1) | Decreased workload |  |
|  |  |  |
| Long COVID (5)  Sick leave (19)  Musculoskeletal pain (8)  Complications due to personal protective equipment (1)  Absence due to care of sick child (1) | Other consequences | Consequences of the COVID-19 pandemic |
| ^a^ Number of participants answering the question.  ^b^ The total number of codes categorized into different subcategories. Each participant’s response could generate several codes and contribute to multiple subcategories forming each category. One category could therefore comprise a higher number of counts than the total number of participants answering the question. | | |

| **Table S6.** Comments regarding how your impacted physical health due to the COVID-19 pandemic affected your work ability the last six months (12-month follow-up) | | |
| --- | --- | --- |
| **n = 23^a^**  **Total number of counts = 34^b^** | | |
| **Subcategory (number of counts)** | **Category** | **Theme** |
| Less energy (1) | Impaired physical ability | Changed physical ability |
| More energy/strength (2)  Happier (1)  Healthier (1) | Improved physical ability |  |
|  |  |  |
| More work (6)  More patients per workday (2)  Physically heavier work (n=2)  Stress (3) | Increased workload | Changed workload |
| Less physical work (1)  Less work (4) | Decreased workload |  |
|  |  |  |
| Long COVID (4)  Musculoskeletal pain (1)  Complications due to personal protective equipment (2)  Absence due to care of sick child (4) | Other consequences | Consequences of the COVID-19 pandemic |
| ^a^ Number of participants answering the question.  ^b^ The total number of codes categorized into different subcategories. Each participant’s response could generate several codes and contribute to multiple subcategories forming each category. One category could therefore comprise a higher number of counts than the total number of participants answering the question. | | |

| **Table S7.** Comments regarding how the COVID-19 impacted your mental health (Baseline) | | |
| --- | --- | --- |
| **n = 305^a^**  **Total number of counts = 598^b^** | | |
| **Subcategory (number of counts)** | **Category** | **Theme** |
| Stress/pressure (62)  Anxiety/worry (78)  Low mood/depression (20)  Fear (2)  Boredom/monotony (7)  Fatigue/lethargy (9)  Tiresome (1)  Feeling burdened/weighed down (3)  Impatience/irritability (1)  Exhaustion (3)  Decreased social motivation (2)  Less resilience (1)  Frustration (4)  Sadness (1)  Nothing to look forward to (1)  Feeling low (2)  Less positive (1)  Hopelessness (1)  Difficult/heavy feelings (1)  Feeling worse (1)  Sleep disturbances (3)  Decreased physical activity  affecting mental well-being (4) | Mental ill health | Mental ill health/consequences |
|  |  |  |
| Social isolation (11)  Longing for group context/social interaction (11)  Fewer social contacts (39)  Affected private relationships (1)  Reduced personal support (1) | Social consequences | Social consequences |
| Fewer positive aspects in daily life (2)  Fewer opportunities for enjoyable leisure (2)  Various facilities being closed (2)  Restricted daily activities (5)  Limited freedom (1) | Restrictions on leisure |  |
|  |  |  |
| Reduced stress (8)  Improved mental health (1)  Reduced performance anxiety (1)  More flexibility in life (1)  Positive/grateful/optimistic (2)  Time for reflection (4)  Grateful/pleased about serving an important function (1)  Slower pace of life (3) | Improved mental health | Positive consequences |
| More family time (1)  Fewer social expectations/contacts (2) | More family time |  |
|  |  |  |
| Fear of infection (75)  Fear/worry about COVID (6)  Worry about the health of loved ones (17)  Concern about long COVID (2)  Uncertainty about long COVID prognosis (1) | Fear of COVID | COVID-related concerns |
| Stress/news anxiety (3)  Grief/sadness about the pandemic (6)  Emotional burden from patients’ fears (3)  Avoiding pandemic-related social media/news (1)  Uncertainty about the future (n=36) | General human concerns |  |
| Inability to work (1)  Less work (2)  Changes in work routines (1)  Increased workload due to recommendations (1)  Reduced job satisfaction (3)  Disrupted routines (3) | Impact on work | Work-related concerns |
| Uncertainty (9)  Financial stress/business or clinic (115) | Work/economy-related worry |  |
|  |  |  |
| Stress about others not following recommendations (1)  Feeling conflicted about adherence to recommendations at work (1)  Constantly thinking about recommendations/behavior (3)  Uncertainty about how manual therapists should act (1) | Conflict between work and recommendations | Double standards |
| ^a^ Number of participants answering the question.  ^b^ The total number of codes categorized into different subcategories. Each participant’s response could generate several codes and contribute to multiple subcategories forming each category. One category could therefore comprise a higher number of counts than the total number of participants answering the question. | | |

| **Table S8.** Comments regarding how the COVID-19 impacted your mental health the last six months (12-month follow-up) | | |
| --- | --- | --- |
| **n = 135^a^**  **Total number of counts count = 205^b^** | | |
| **Subcategory (number of counts)** | **Category** | **Theme** |
| Stress/pressure (16)  Anxiety/worry (11)  Low mood/depression (12)  Boredom/monotony (3)  Fatigue/lethargy (6)  Tiresome (3)  Feeling burdened/weighed down (1)  Impatience/irritability (1)  Exhaustion (6)  Frustration (1)  Hopelessness (1)  Sleep disturbances (1)  Loneliness (2)  Feeling defeated (2)  Suicidal thoughts (1) | Mental ill health | Mental ill health/consequences |
| Long COVID (2)  Sick leave (2)  Vaccine side effects (1) | Illness |  |
|  |  |  |
| Social isolation (4)  longing for group context/social interaction (2)  Fewer social contacts (11)  Affected private relationships (1)  Reduced personal support (2) | Social consequences | Social consequences |
| Fewer opportunities for enjoyable leisure (1)  Limited freedom (4) | Restrictions on leisure |  |
|  |  |  |
| Reduced stress (1)  Time for reflection (1)  Hopeful/positive (1)  Feeling safe/calm due to vaccination (4)  Work satisfaction (3)  Relaxed (1)  Present (1) | Improved mental health | Positive consequences |
| Fewer social expectations/contacts (1) | More time with family |  |
|  |  |  |
| Fear of infection (12)  Worry about the health of loved ones (5) | Fear of COVID | COVID-related concerns |
| Stress/anxiety from news coverage (2)  Emotional burden from patients’ fears (2)  Hearing patients constantly talk about COVID (1)  Uncertainty about the future (8) | General human concerns |  |
| Less work (1)  Changes in work routines (1)  Increased workload (2)  Additional workload due to recommendations (5)  Reduced job satisfaction (5)  Job termination (1)  Business closure (1) | Impact on work | Work-related concerns |
| Uncertainty (2)  Financial stress (business/clinic) (38) | Work and financial concerns |  |
|  |  |  |
| Stress about others not following recommendations (1)  Double standards in following recommendations at work (1)  Constantly thinking about recommendations/behavior (2)  Skepticism toward pandemic management (3)  Pressure to vaccinate (1)  Worry about restrictions and recommendations (5)  Fatigue from restrictions (2) | Conflict between work and recommendations | Double standards |
| ^a^ Number of participants answering the question.  ^b^ The total number of codes categorized into different subcategories. Each participant’s response could generate several codes and contribute to multiple subcategories forming each category. One category could therefore comprise a higher number of counts than the total number of participants answering the question. | | |

| **Table S9.** Comments regarding how your impacted mental health due to the COVID-19 pandemic affected your work ability (Baseline) | | |
| --- | --- | --- |
| **n = 43^a^**  **Total number of counts = 54^b^** | | |
| **Subcategory (number of counts)** | **Category** | **Theme** |
| Fatigue/exhaustion (9)  Reduced energy/stamina (2)  Decreased motivation to work (3)  Reduced well-being (2)  Feeling defeated (1)  Low mood/depression (1)  Reduced joy/interest in life (2)  Apathy (1) | Burnout/depression | Negative psychological consequences |
| Fear (1)  Anxiety/worry (5)  Stress about infection (3)  Stress (7)  Uncertainty (1)  Emotional burden from patients' mental ill health (1) | Anxiety/worry |  |
| Reduced work hours (1)  Reduced work assignments (2)  Inadequate work performance (1) | Impact on work (quantity/quality) |  |
| Distracted (1)  Poor memory (2) | Cognitive consequences |  |
| Improved focus (1)  Reduced stress (2)  Increased energy/stamina (3)  Greater joy (1)  Time to address tasks (1) | Positive consequences | Positive consequences |
| ^a^ Number of participants answering the question.  ^b^ The total number of codes categorized into different subcategories. Each participant’s response could generate several codes and contribute to multiple subcategories forming each category. One category could therefore comprise a higher number of counts than the total number of participants answering the question. | | |

| **Table S10.** Comments regarding how your impacted mental health due to the COVID-19 pandemic affected your work ability the last six months (12-month follow-up) | | |
| --- | --- | --- |
| **n = 29^a^**  **Total number of counts = 50^b^** | | |
| **Subcategory (number of counts)** | **Category** | **Theme** |
| Fatigue/exhaustion (5)  Reduced energy/stamina (2)  Decreased motivation to work (5)  Reduced well-being (1)  Low mood/depression (1)  Sleep problems (2)  Difficulty maintaining a positive mood (2)  Lack of joy (1)  Feeling mentally unwell (2)  Unable to go to work (1)  Poor recovery (1)  Sick leave (2) | Burnout/depression | Negative psychological consequences |
| Stress (3)  Uncertainty (1) | Stress/worry |  |
| Reduced work hours (7)  Inadequate work performance (1)  Continued working despite challenges (1)  No vacation (1)  Fewer patients (2) | Impact on work (quantity/quality) |  |
| Reduced focus (2)  Cognitive impairments (1) | Cognitive consequences |  |
| Greater joy (1)  Feeling relaxed (1)  Increased creativity (1)  Improved job functioning (2)  Better stress management (1) | Positive consequences | Positive consequences |
| ^a^ Number of participants answering the question.  ^b^ The total number of codes categorized into different subcategories. Each participant’s response could generate several codes and contribute to multiple subcategories forming each category. One category could therefore comprise a higher number of counts than the total number of participants answering the question. | | |

| **Table S11.** List the three main worries related to your clinical practice and the COVID-19 pandemic* (6 month follow-up) | | |
| --- | --- | --- |
| **n = 373^a^**  **Total number of counts = 787^b^** | | |
| **Subcategory (number of counts)** | **Category** | **Theme** |
| Staff safety (2) | Colleague safety | Worry about infection |
| Worry when vulnerable groups book appointments (2)  Infection risk for vulnerable groups (14)  Treating vulnerable groups (6) | Patient safety |  |
| When patients do not use protective equipment (1)  When patients do not follow recommendations (4)  Patients not being entirely honest about symptoms (3)  Long COVID (9)  Being last of healthcare workers to get vaccinated (3)  Being pregnant during the pandemic (2) | Personal safety |  |
| Worry about infection (self/patient/colleagues) (190)  Asymptomatic infection (47)  Worry about not being able to maintain distance (28)  Carrying infection to family/relative/friends (18)  Spending a long time with the same person in the same room (6)  Worry about becoming a "super-spreader" (3)  Patients with cold symptoms (1) | Worry about infection |  |
| Economy (161)  Less patient appointments (63)  Closing down due to drop in patient numbers (6)  Getting ill and being unable to work (32)  Sick Personnel (8)  Closing down due to infection (14)  Closing down due to restrictions (48)    Bankruptcy (11)  Being unemployed (10)  Worry relating to absence due to care of sick child (1)  Personnel on long-term sick-leave (3)  Not getting company assignments again (4)  New waves of infection (1)  Personal economy (6)  Infection leading to a bad reputation (8)  Change profession (5) | Economic worry | Economic worry |
| The pandemic never ends (1)  Uncertainty concerning the development of the pandemic (6)  More pandemics (1)  Society dissolves (1)  Unable to plan (3)  General worry (3) | Existential worry | Existential worry |
| Illogical handling of the pandemic (2)  Unclear/uncertain information/recommendations (4)  How to handle protective routines (2)  Not fulfilling requirements for safety (6)  How long to maintain restrictions (1)  Official recommendations (13) | Uncertainty about recommendations | Uncertainty about recommendations |
| worry about mental health of patients (3)  Patients not seeking care when in need (18)  Quality of care (2)  Patients not seeking care due to fear of infection (1) | Concern for patients’ health | Concern for patients’ health |
| ^a^ Number of participants answering the question.  ^b^ The total number of codes categorized into different subcategories. Each participant’s response could generate several codes and contribute to multiple subcategories forming each category. One category could therefore comprise a higher number of counts than the total number of participants answering the question.  *In the header of the section it was specified that the question related to the last six months. | | |

| **Table S12.** List the three main worries related to your clinical practice and the COVID-19 pandemic* (12 month follow-up) | | |
| --- | --- | --- |
| **n = 380^a^**  **Total number of counts = 902^b^** | | |
| **Subcategory (number of counts)** | **Category** | **Theme** |
| Staff safety (11)  Mental health of colleagues (2) | Colleague safety | Worry about infection |
| Infection risk for vulnerable groups (15)  Treating vulnerable groups (7) | Patient safety |  |
| When patients do not use protective equipment (2)  When patients do not follow recommendations (4)  Patients not being entirely honest about symptoms (5)  Long COVID (24)  Being pregnant during the pandemic (2)  Those vaccinated not following recommendations (2)  Vaccine side effects (2)  Vaccine not providing enough protection (3) | Personal safety |  |
| Worry about infection (self/patient/colleagues) (183)  Asymptomatic infection (27)  Worry about not being able to maintain distance (18)  Patients with cold symptoms (3)  Worry about becoming a "super-spreader" (2)  Carrying infection to family/relative/friends (14) | Worry about infection |  |
| Economy (160)  Less patient appointments (123)  Closing down due to drop in patient numbers (7)  Getting ill and being unable to work (52)  Closing down due to infection (6)  Closing down due to restrictions (37)  Bankruptcy (10)  Being unemployed (13)  Worry relating to absence due to care of sick child (4)  Personnel on long-term sick leave (6)  Not getting company assignments again (1)  New waves of infection (5)  Personal economy (15)  Infection leading to a bad reputation (4)  Change profession (1)  No financial support (4)  Unable to pursue further education (2) | Economic Worry | Economic Worry |
| The pandemic never ends (1)  Uncertainty concerning the development of the pandemic (9)  More pandemics (1)  Unable to plan (1)  General worry (1) | Existential Worry | Existential Worry |
| Illogical handling of the pandemic (5)  Unclear/uncertain messages/recommendations (2)  Not fulfilling requirements for safety (15)  Official recommendations (22)  Requirements for vaccines/vaccine passports (12)  Being accused of not following recommendations (12)  Whether the protective equipment is sufficient (5)  Censorship, propaganda from the Public Health Agency (4) | Uncertainty about recommendations | Uncertainty about recommendations |
| worry about the mental health of patients (2)  Patients not seeking care when in need (8)  Quality of care (1)  Patients not seeking care due to fear of infection (22)  Having to treat vaccine side effects (2)  Isolation leading to mental health issues (1) | Concern for patients’ health | Concern for patients’ health |
| ^a^ Number of participants answering the question.  ^b^ The total number of codes categorized into different subcategories. Each participant’s response could generate several codes and contribute to multiple subcategories forming each category. One category could therefore comprise a higher number of counts than the total number of participants answering the question.  *In the header of the section it was specified that the question related to the last six months. | | |
