# Supplementary material for: Health aspects and lifestyle of licensed manual therapists during the COVID-19 pandemic in Sweden: The CAMP cohort study
Source: PLoS One. 2025 Oct 7;20(10):e0327600. doi: 10.1371/journal.pone.0327600 (PMC12503269; doi:10.1371/journal.pone.0327600)
Supplement: Attachment — Submitted filename: PONE-D-24-54978-review.pdf [file pone.0327600.s002.pdf]

Health aspects and lifestyle of licensed manual therapists during the COVID-19 pandemic in Sweden; the CAMP cohort study, by Weiss et al.

I am grateful for the opportunity to contribute to the peer review process of this manuscript, which refers to a longitudinal study in a COVID management context different from the ordinary one of alternating lockdowns.

The authors have presented very clearly the purpose of their study, the methods used and the results obtained. I would like to express my sincere appreciation to them for the work done. Below are some minor comments and some ideas for a deeper understanding of the study results.

1. In the Introduction the authors should add information about vaccination in Sweden. According to Spetz et al., in Sweden, the vaccination program started on 27th December 2020 and by 5th November 2021, 85% of the adult population had received their first dose. Therefore, the baseline data should refer almost entirely to subjects who were not yet vaccinated, while the follow-up data were collected when the vaccine was fully available and, at 12 months, after having also overcome the major uncertainties associated with vaccination. In my opinion, this aspect should also be considered in the discussion.

2. sub-section Physical activity: If I am not mistaken, according to the cited reference [18], the physical activity data should have been managed in a categorical way, on a scale ranging from 3 to 18 instead of the proposed “minutes of activity”. Instead, the authors considered the minutes, converting the categorical response into the central numerical value of the class. It is not clear to me, therefore, whether this method of converting categorical data into a continuous variable (minutes) has had any validation in the literature. As a small comment, regarding the response alternatives: “1-30 minutes”, “30-60 minutes”, “60-90 minutes”, etc., a more accurate data collection would be obtained, for example, by indicating "1-29 minutes", "30-59 minutes" and so on. The original indication "Less than 30 minutes" (as reported in [18]) would be preferable to "1-30", in my opinion.

3. At the end of the Tobacco and alcohol use section there is a typo on AUDIT-C.

4. Regarding the free text variables, how was the question formulated at 12 months? Such as baseline (What have you done to promote your own health during the COVID-19 pandemic?) or, for example, "What have you done to promote your own health during the last 12 months?".

5. Please add appropriate citations for R packages gee and multgee.

6. sub-section Longitudinal lifestyle patterns: I believe that indicating the sedentary time variation even in minutes can help the reader.

7. Table 2: Why was the 34-year threshold taken into consideration? I suggest adding a reason. Furthermore, to assist readers who are not experts in GEE methods, I suggest clarifying what is reported in the line "Mean difference in change baseline to 12 months (95% CI)". The same goes for other similar tables.

8. Table 5: To highlight the appearance of the new category "Change profession", I suggest introducing a decimal instead of reporting 0%. Personally, I was very intrigued by the classification of "Change profession" among the activities for the promotion of mental health. Did the respondents themselves give this indication? Since it seems to be a relevant result, perhaps the authors can spend a few more words on it.

9. Table 6 and related text (lines 233-237): If I understand correctly, all 202 individuals who responded to the baseline indicated at least one negative impact, but 70 of them also indicated positive impacts. Is that right?

10. Tables 5-10 overall: I would like to suggest reviewing the indication of percentages. With reference to table 10, it is really confusing to find indications higher than 100%. Perhaps, these particular cases could be better explained in the legend. Or, the analysis procedure of the individual responses could be illustrated in greater detail in the methods, perhaps with an example (even a fictitious one). Finally, why COVID-19-related worries were not asked at the baseline as well?

References

Spetz, M., Lundberg, L., Nwaru, C., Li, H., Santosa, A., Leach, S., ... & Nyberg, F. (2022). The social patterning of Covid-19 vaccine uptake in older adults: A register-based cross-sectional study in Sweden. The Lancet Regional Health–Europe, 15.
